# Murine breast cancer feed arteries are thin-walled with reduced α_1A_-adrenoceptor expression and attenuated sympathetic vasocontraction

**DOI:** 10.1186/s13058-018-0952-8

**Published:** 2018-03-22

**Authors:** Anne Sofie Froelunde, Marit Ohlenbusch, Kristoffer B. Hansen, Nicolai Jessen, Sukhan Kim, Ebbe Boedtkjer

**Affiliations:** Department of Biomedicine, Aarhus University, Ole Worms Allé 3, building 1170, DK-8000 Aarhus C, Denmark

**Keywords:** Adrenergic receptors, Arterial structure, Breast cancer, Sympathetic nerve activity, Tumor microenvironment

## Abstract

**Background:**

Perfusion of breast cancer tissue limits oxygen availability and metabolism but angiogenesis inhibitors have hitherto been unsuccessful for breast cancer therapy. In order to identify abnormalities and possible therapeutic targets in mature cancer arteries, we here characterize the structure and function of cancer feed arteries and corresponding control arteries from female FVB/N mice with ErbB2-induced breast cancer.

**Methods:**

We investigated the contractile function of breast cancer feed arteries and matched control arteries by isometric myography and evaluated membrane potentials and intracellular [Ca^2+^] using sharp electrodes and fluorescence microscopy, respectively. Arterial wall structure is assessed by transmission light microscopy of arteries mounted in wire myographs and by evaluation of histological sections using the unbiased stereological disector technique. We determined the expression of messenger RNA by reverse transcription and quantitative polymerase chain reaction and studied receptor expression by confocal microscopy of arteries labelled with the BODIPY-tagged α_1_-adrenoceptor antagonist prazosin.

**Results:**

Breast cancer feed arteries are thin-walled and produce lower tension than control arteries of similar diameter in response to norepinephrine, thromboxane-analog U46619, endothelin-1, and depolarization with elevated [K^+^]. Fewer layers of similarly-sized vascular smooth muscle cells explain the reduced media thickness of breast cancer arteries. Evidenced by lower media stress, norepinephrine-induced and thromboxane-induced tension development of breast cancer arteries is reduced more than is explained by the thinner media. Conversely, media stress during stimulation with endothelin-1 and elevated [K^+^] is similar between breast cancer and control arteries. Correspondingly, vascular smooth muscle cell depolarizations and intracellular Ca^2+^ responses are attenuated in breast cancer feed arteries during norepinephrine but not during endothelin-1 stimulation. Protein expression of α_1_-adrenoceptors and messenger RNA levels for α_1A_-adrenoceptors are lower in breast cancer arteries than control arteries. Sympathetic vasocontraction elicited by electrical field stimulation is inhibited by α_1_-adrenoceptor blockade and reduced in breast cancer feed arteries compared to control arteries.

**Conclusion:**

Thinner media and lower α_1_-adrenoceptor expression weaken contractions of breast cancer feed arteries in response to sympathetic activity. We propose that abnormalities in breast cancer arteries can be exploited to modify tumor perfusion and thereby either starve cancer cells or facilitate drug and oxygen delivery during chemotherapy or radiotherapy.

**Electronic supplementary material:**

The online version of this article (10.1186/s13058-018-0952-8) contains supplementary material, which is available to authorized users.

## Background

Breast cancer is the most common non-cutaneous cancer form in the Western world with more than 250,000 annual new cases in the USA alone. Around one in eight women develop invasive breast cancer over the course of life.

Increased proliferative rate of breast cancer cells necessitates high metabolic activity, which again requires a constant supply of metabolic substrates and O_2_. High O_2_ utilization rates and insufficient blood supply lead to inadequate tissue oxygenation at least in micro-areas of human breast cancer tissue [1]. As evidenced by association between tumor perfusion and breast cancer O_2_ consumption [1], O_2_ availability limits oxidative metabolism in cancer tissue, which supports the anti-cancer potential of interventions that manipulate O_2_ and nutrient delivery to tumors.

The clinical impact of anti-angiogenic drugs used to target generation of breast cancer arteries has been disappointing [2]. Modifying tumor perfusion by targeting functions of already formed tumor arteries provides an attractive alternative: selective vasoconstriction of cancer arteries will lower tumor perfusion and starve cancer cells; and if sufficiently pronounced, blood flow arrest can cause tumor infarction as observed in response to thrombus induction in tumor blood vessels [3]. Selective vasodilation of cancer arteries during treatment periods also has therapeutic potential by facilitating delivery of chemotherapeutic agents and enhancing radiotherapeutic responses through elevated local O_2_ pressures [4]. Delivery of drugs to cancer tissue is challenged by irregular flow patterns [5]: large variations in blood flow between tumor regions result in inhomogeneous drug delivery; and even in individual vessel segments, blood flow is temporally variable with blood flow periodically stopping or reversing. Controlling precapillary resistance is also important for fluid exchange, which again plays a role in extravasation and shunting of drugs.

The heterogeneous and typically insufficient blood supply to cancer tissue creates local hypoxia and accumulation of acidic waste products. The extracellular microenvironment of breast cancer tissue is fundamentally different from normal tissue and contains, for instance, high concentrations of H^+^, ATP, and paracrine signaling factors (e.g., endothelin and vascular endothelial growth factor) [6]. These local biochemical disturbances are hostile for most normal cells including those of the immune system [7, 8]. When the metabolic stress of the tumor microenvironment remains within the limits of cancer cell survival, it provides a survival advantage for cancer cells compared to normal cells and constitutes a selection pressure that favors cancer cells with more malignant phenotypes [9]. Targeted vasodilation of cancer arteries may relieve intermittent blood flow patterns and provide more uniform blood distributions that can improve the typical mismatch between blood flow and metabolism, minimize disturbances in local environment, inhibit upregulation of hypoxia-inducible genes, and boost the ability of the immune system to recognize and fight cancer cells.

The architecture of the cancer vasculature is distorted with leaky and disorganized blood vessels sharing characteristics of arterioles, capillaries, and venules [10]. Variable density of contractile elements challenges interventions to substantially modify tumor perfusion. Rather than studying dysmorphic intratumoral blood vessels, we here focused on feed arteries that supply cancer tissue with blood, yet in their overall wall composition resemble normal arteries and thus are expected to maintain ability for considerable tension development.

In the current study, we explored the structure and function of breast cancer feed arteries from mice with ErbB2-induced breast cancer. ErbB2 is an orphan growth factor receptor that facilitates breast carcinogenesis: ~ 20% of human breast cancers show overexpression or gene amplification of ErbB2, and targeted treatment—e.g., with the functional monoclonal antibody trastuzumab—improves the prognosis [11]. We tested the hypothesis that breast cancer feed arteries are functionally and structurally distinct from equivalent control arteries and find that breast cancer arteries (a) are thin-walled with overall lower capacity for vasocontraction and (b) display deregulation of adrenergic and thromboxane-mediated signaling leading to reduced perivascular, nerve-mediated, contractile responses. We propose that attenuated constriction of breast cancer feed arteries enhances tumor perfusion and that preferential changes in tumor vascular resistance relative to other vascular beds are therapeutically attainable.

## Methods

We isolated breast cancer feed arteries and matched control arteries from female virgin FVB mice (FVB/N-Tg(MMTVneu)202Mul/J; Jackson Laboratories, ME, USA) with breast epithelial overexpression of ErbB2—also known as neu and HER2—under transcriptional control of the mouse mammary tumor virus promoter [12]. Mice were housed in the animal facility at the Department of Biomedicine, Aarhus University, under a 12-h light/12-h dark cycle with free access to food and water. ErbB2-overexpressing mice develop macroscopically identifiable breast cancer with median latency of 205 days [12], and they were palpated weekly for tumor detection. Cancer feed arteries were adherent to breast carcinomas that developed along the mammary lines extending from the axilla to the inguen; control arteries were similarly located anatomically but distanced at least 3 cm from macroscopically identifiable tumor tissue. All animal handling was approved by the Danish Animal Experiments Inspectorate (2012-15-2935-00002) and performed according to the guidelines from Directive 2010/63/EU of the European Parliament on the protection of animals used for scientific purposes.

### Small artery myography

Mice were euthanized by cervical dislocation or CO_2_ inhalation, and breast cancer feed arteries and matched control arteries isolated under a stereomicroscope. Arteries were mounted on 40-μm stainless steel wires for isometric myography in multi-channel myograph chambers (610 M; DMT, Denmark) filled with Ca^2+^-free physiological saline solution (PSS, see composition below) in order to induce full relaxation and avoid stretch-induced damage. Myograph chambers were then washed with standard PSS and heated to 37 °C. Breast cancer feed arteries and matched control arteries had very similar internal diameters (171 ± 5 μm *vs.* 178 ± 6 μm; *n* = 56; *P* = 0.32; paired two-tailed Student’s *t* test) when normalized to 90% of the internal diameter corresponding to a transmural pressure of 100 mmHg [13]. Cancer and control arteries from a given mouse were both excluded from further analyses if either artery developed less than 1 mN force in response to application of 63.9 mmol/L extracellular (o) K^+^. Data were collected in LabChart Pro (ADInstruments, New Zealand).

As per previous experience with murine arteries [14], vasocontractile responses to endothelin-1, norepinephrine, thromboxane analog U46619, and depolarization with elevated [K^+^]_o_ were tested through cumulative applications. Endothelium-dependent vasorelaxation to acetylcholine was evaluated as single doses in order to avoid spontaneous relaxations or tachyphylaxis in arteries pre-contracted with norepinephrine. Experiments involving elevated [K^+^]_o_ were performed in the presence of 1 μmol/L of the non-selective α-adrenoceptor antagonist phentolamine in order to avoid the effects of norepinephrine released from perivascular nerve endings in response to depolarization.

The PSS contained (in mmol/L) 140 Na^+^, 4 K^+^, 1.6 Ca^2+^, 1.2 Mg^2+^, 124 Cl^−^, 22 HCO_3_^−^, 1.2 SO_4_^2−^, 1.2 H_2_PO_4_^−^, 10 HEPES, 5.5 glucose, and 0.03 ethylenediaminetetraacetic acid (EDTA); pH was adjusted to 7.4 when gassed with 5% CO_2_/balance air at 37 °C. In experiments mimicking metabolic acidosis, pH of the bath solution was reduced to 6.8 by lowering HCO_3_^−^ to 5.5 mmol/L through substitution with Cl^−^. In Ca^2+^-free PSS, the 1.6 mmol/L Ca^2+^ and associated 3.2 mmol/L Cl^−^ were omitted.

### Measurements of intracellular [Ca^2+^]

We evaluated intracellular [Ca^2+^] in vascular smooth muscle cells (VSMCs) using fluorescence microscopy based on the Ca^2+^-sensitive fluorophore Fura-2 (Life Technologies, CA, USA) [15]. Arteries mounted in single-channel wire myographs (320A; DMT) were loaded with 5 μmol/L of the acetoxymethyl ester form of Fura-2 for 2 × 30 min at 37 °C and investigated using a Leica DM IRB inverted microscope (Germany) with a ×20 objective (numerical aperture 0.5) connected to a PTI DeltaScan fluorescence system (NJ, USA). Arteries were excited alternately at 340 and 380 nm and emission light collected around 530 nm. Background fluorescence prior to loading was subtracted from recorded emissions before ratio (340/380) calculation. Relative changes in Fura-2 fluorescence were calculated for each artery and normalized to the average response in control arteries.

### Membrane potential measurements

Arteries were mounted in a customized, single-channel, water-heated wire myograph (DMT). Membrane potential (V_m_) was measured using aluminum silicate microelectrodes (World Precision Instruments, UK; resistance ~40–120 MΩ when backfilled with 3 mol/L KCl) coupled to an Intra-767 amplifier (World Precision Instruments), visualized on an oscilloscope (Gould-Nicolet Technologies, UK), and continuously stored with a PowerLab system (ADInstruments) [16]. Electrode entries into cells resulted in abrupt drops in voltage followed by sharp returns to baseline upon retraction [17].

### Artery structure and stereological analyses

Arterial wall structure was assessed based on transmission light microscopy of arteries mounted in dual-channel wire myographs (420A; DMT) with glass windows in the chamber base [18]. Media thickness was measured under gentle stretch using a filar micrometer at six individual points for each artery [19]. To determine the relationship between arterial wall structure and tumor size, we calculated the volumes of excised tumors based on the formula:

$$ \mathrm{Volume}=\mathrm{Length}\times \mathrm{Width}\times \mathrm{Depth}\times \uppi /6 $$

After normalization, arteries were fixed for 20 min in 4% paraformaldehyde and stored in phosphate-buffered saline at 5 °C. Paraffin-embedded arteries cut longitudinally—i.e., perpendicular to the long axis of the VSMCs—into 3-μm sections were stained with Giemsa or hematoxylin and eosin. Through identification of nuclear ends—i.e., nuclei present in one section but absent in the neighboring section—the numerical cellular density and VSMC dimensions were calculated for the media volume demarcated by two consecutive sections based on the unbiased stereological disector method [18, 20, 21]. We calculated media stress—as wall tension divided by media thickness—in order to evaluate if differences in contractile function are explained by structural variation between the arteries.

### Electrical field stimulation

Perivascular nerve endings of arteries mounted in dual-channel wire myographs (420A; DMT) were stimulated using bipolar electrical fields created between 200-pm thick platinum electrodes (DMT). Single trains of 10-s duration consisting of 0.1-ms square-wave pulses of 35 mA with frequencies from 0.5 to 64 Hz were delivered by a CS200 current stimulator (DMT). Dependency on nerve conduction was confirmed with 1 μmol/L of the specific voltage-gated Na^+^-channel inhibitor tetrodotoxin (TTX) [22]. Prazosin was applied in order to test involvement of α_1_-adrenoceptors [23, 24].

### Labelling of α_1_-adrenergic receptors

We evaluated expression of α_1_-adrenergic receptors using the boron dipyrromethene (BODIPY™)-labelled α_1_-adrenoceptor antagonist prazosin [25]. Freshly dissected arteries incubated with 1 μmol/L BODIPY™ FL prazosin (B7433; ThermoFisher Scientific, MA, USA) for 90 min at room temperature in the dark were investigated by confocal microscopy: arteries were excited at 488 nm and emission light collected at wavelengths between 505 and 530 nm. Z-stack image series of 1-μm optical steps were constructed and the second image within the media used for quantification. Strong autofluorescence of the internal elastic lamina defines the transition between tunica media and tunica intima and ensures that the confocal planes of quantified images are at comparable levels in the media. Pixels with fluorescence intensities above a pre-selected threshold (2000 arbitrary units) were considered positive. Labeled areas were expressed relative to the total area of the optical sections covered by tunica media. Essentially no fluorescence was observed under the experimental conditions when no BODIPY™-labelled prazosin was added (data not shown).

### Reverse transcription and quantitative PCR analyses

Expression of messenger RNA for α_1_-adrenergic receptors was evaluated by reverse transcription and quantitative PCR analyses. Commercially available primers and probes (TaqMan® Gene Expression Assays; Applied Biosystems, CA, USA) against α_1A_- (Mm00442668_m1), α_1B_- (Mm00431685_m1), and α_1D_-adrenoceptors (Mm01328600_m1) were used with the transferrin receptor (Mm01344478_m1) serving as reference. Isolated arteries were disrupted in lysis buffer using Qiagen TissueLyser (Denmark). Total RNA was isolated with the RNeasy Micro QiaCube kit and—after DNase treatment—reverse transcribed using Reverse Transcriptase III (Invitrogen, CA, USA), RNase inhibitor Superase (Invitrogen), and random decamer primers (Eurofins Genomics, Germany). To control for genomic amplification, RT– experiments were performed without reverse transcriptase added. PCRs performed with a Stratagene MX3000P qPCR system (AH Diagnostics, Denmark) consisted of 1 cycle at 95 °C for 4 min followed by 50 cycles at 92 °C for 10 s, 55 °C for 20 s, and 72 °C for 30 s. Expression levels for α_1_-adrenoceptors in cancer arteries relative to control arteries were calculated based on the 2^–ΔΔCt^ method [26].

### Statistics

Data are expressed as mean ± SEM; *n* indicates the number of mice. *P* < 0.05 was considered statistically significant. We used the paired two-tailed Student’s *t* test to compare one variable in cancer and matched control arteries. Arteries from different mice were compared using the unpaired two-tailed Student’s *t* test. The one-sample *t* test was used to compare single distributions to hypothetical means. We evaluated the effects of two variables on the measured variable, measured multiple times for each mouse, using repeated measures two-way analysis of variance (ANOVA) followed by the Sidak or Bonferroni post-hoc test. Dependency of media structure on tumor volume was tested by least-squares linear regression analyses. Concentration-response relationships were fitted to sigmoidal functions and compared with extra-sum-of-squares *F* tests. Right-skewed data were log-transformed to achieve normal distribution. Statistical analyses were performed using GraphPad Prism 7.03 software.

## Results

We explored the function and structure of breast cancer feed arteries and equivalent control arteries based on a murine model of ErbB2-induced breast cancer. Breast cancer feed arteries were adherent to breast cancer tissue whereas control arteries of comparable diameter were from corresponding anatomical locations along the mammary lines but situated at least 3 cm from macroscopically identifiable tumor tissue.

### Vasocontraction is attenuated in breast cancer feed arteries

**Fig. 1:**
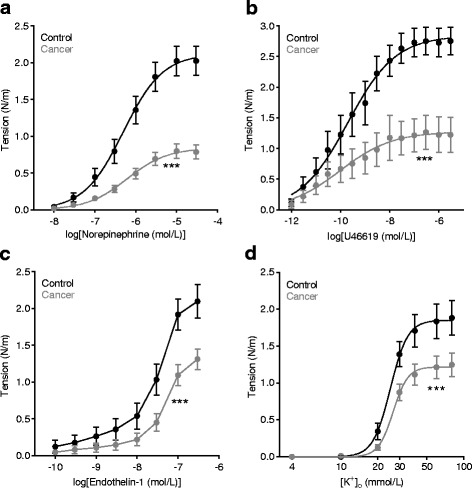
Vasocontraction of breast cancer feed arteries is attenuated compared to matched control arteries. Concentration-dependent contractions in response to norepinephrine (**a**) (*n* = 32), U46619 (**b**) (*n* = 12), endothelin-1 (**c**) (*n* = 19), and elevated extracellular K^+^ ([K^+^]_o_) (**d**) (*n* = 16) at pH_o_ 7.4. Experiments with elevated [K^+^]_o_ were performed in the presence of 1 μmol/L phentolamine in order to avoid the effects of norepinephrine released from sympathetic nerve endings in response to depolarization. Data were fitted to sigmoidal functions using least-squares regression analyses and curves compared by extra-sum-of-squares *F* tests. ***P < 0.001 *vs.* control

Breast cancer feed arteries responded with reduced vasocontraction compared to control arteries when exposed to norepinephrine (Fig. 1a), thromboxane analog U46619 (Fig. 1b), endothelin-1 (Fig. 1c), or increased [K^+^]_o_ (Fig. 1d). Elevated [K^+^]_o_ was applied to depolarize and contract VSMCs independently of receptor activation; and these experiments were performed in the presence of 1 μmol/L phentolamine in order to avoid the effects of norepinephrine released from perivascular nerve endings in response to depolarization.

The extracellular compartment of solid cancer tissue is characteristically acidic and may reach as much as one pH unit below values typical for normal tissue [27]. We therefore investigated whether reduced vasocontraction of breast cancer feed arteries was also observed under acidic conditions. As expected, contraction of control arteries was attenuated at pH_o_ 6.8 compared to pH_o_ 7.4 (Additional file 1: Figure S1). Furthermore, responses to norepinephrine (Additional file 1: Figure S1A), endothelin-1 (Additional file 1: Figure S1B), and depolarization with elevated [K^+^]_o_ (Additional file 1: Figure S1C) were lower in cancer feed arteries compared to control arteries also at pH_o_ 6.8.

### Breast cancer feed arteries are thin-walled

**Fig. 2:**
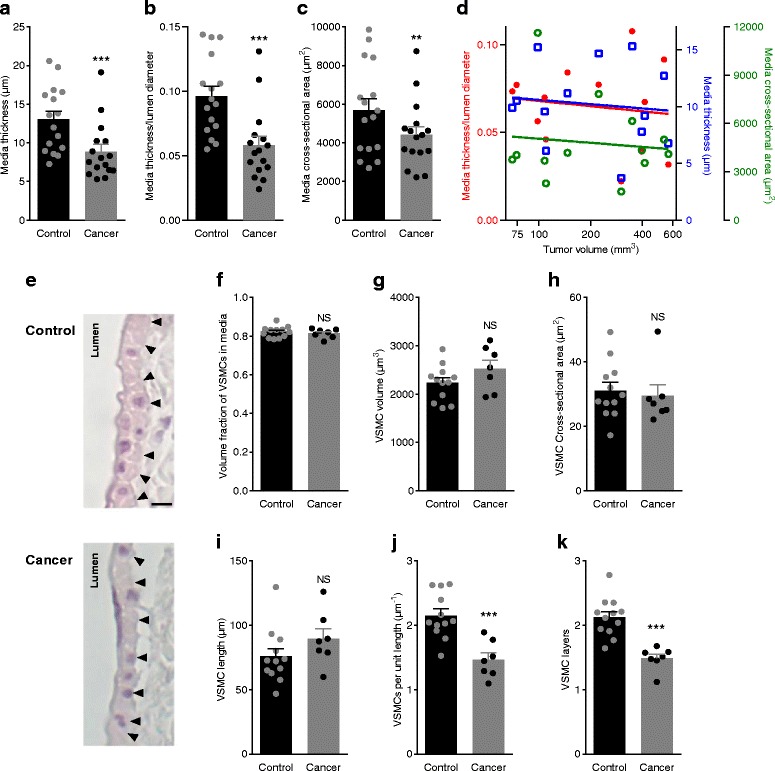
Breast cancer feed arteries are thin-walled compared to control arteries because of fewer vascular smooth muscle cells (VSMCs) in the tunica media. **a**-**c** Media thickness (**a**), media thickness/luminal diameter ratio (**b**), and media cross-sectional area (**c**) (*n* = 16). **d** Media thickness, media thickness/luminal diameter ratio, and media cross-sectional area of breast cancer feed arteries from a separate experimental series plotted as function of tumor volume (*n* = 13). The slopes of the best-fit linear functions did not differ significantly from zero: *P* = 0.71, 0.67, and 0.72, respectively. **e** Histological images representative of the wall structure in control arteries (upper panel) and breast cancer feed arteries (lower panel). Scale bar represents 10 μm; images are shown at the same magnification. The arrowheads demarcate the outer border of the media. **f** Volume fraction of VSMCs in the arterial media (*n* = 7–12). **g**-**i**. Volume (**g**), cross-sectional area (**h**), and length (**i**) of VSMCs (n = 7-12). **j**-**k** Number of VSMCs per unit artery length (**j**) and number of VSMC layers (**k**) (n = 7–12). Data were compared by the paired or unpaired two-tailed Student’s *t* test as appropriate, except for **d** where least-squares linear regression analyses were performed. **P < 0.01, ***P < 0.001. NS: not significantly different *vs.* control

Reduced vasocontractile responses to agonist stimulation (Fig. 1a-c and Additional file 1: Figure S1A,B) and K^+^-induced VSMC depolarization (Fig. 1d and Additional file 1: Figure S1C) are consistent with thinner wall structure. To investigate whether the wall structure of breast cancer feed arteries differs from that of control arteries, we next measured arterial media thickness by transmission light microscopy: media thickness (Fig. 2a) and media thickness to lumen diameter ratio (Fig. 2b) were both reduced in breast cancer feed arteries compared to control arteries as was media cross-sectional area (Fig. 2c). In order to investigate at what stage of breast cancer development and expansion cancer feed arteries attain their altered wall structure, we assessed in a separate series of experiments the media structure of cancer feed arteries from tumors of different sizes (Fig. 2d). Arterial media thickness, media thickness to lumen diameter ratio, and media cross-sectional area were independent of tumor volume in the investigated range between 70 and 600 mm^3^, indicating that the altered arterial structure is an early characteristic of cancer development (Fig. 2d). Based on the unbiased stereological disector technique, we analyzed histological sections (Fig. 2e) and found that the difference in media thickness between breast cancer feed arteries and control arteries was not explained by smaller VSMCs (Fig. 2g-i) but rather by fewer VSMCs per unit artery length (Fig. 2j), which resulted in fewer VSMC layers (Fig. 2k). The fraction of tunica media made up of VSMCs relative to extracellular space did not differ between breast cancer feed arteries and control arteries (Fig. 2f).

### Structure-dependent and structure-independent differences in artery tone

**Fig. 3:**
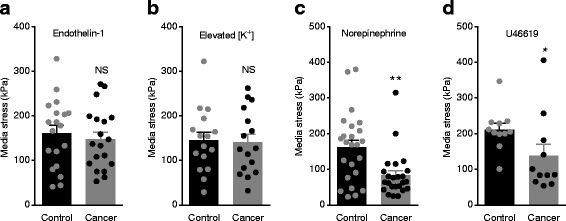
Media stress of breast cancer feed arteries is reduced compared to matched control arteries in response to norepinephrine and U46619 but not in response to endothelin-1 or depolarization with elevated extracellular K^+^ concentration ([K^+^]_o_). Media stress (wall tension divided by media thickness) during stimulation with 300 nmol/L endothelin-1 (**a**) (n = 19), 80 mmol/L K^+^_o_ (**b**) (n = 16), 30 μmol/L norepinephrine (**c**) (*n* = 24), and 3 μmol/L U46619 (**d**) (*n* = 11). Experiments with elevated [K^+^]_o_ were performed in the presence of 1 μmol/L phentolamine in order to avoid the effects of norepinephrine released from sympathetic nerve endings in response to depolarization. Data were compared by the paired two-tailed Student’s *t* test. Responses to U46619 were log-transformed before statistical comparison to achieve normal distribution. *P < 0.05, **P < 0.01, NS: not significantly different *vs.* control

The thinner tunica media contributes substantially to explaining the reduced vasocontractile responses of breast cancer feed arteries compared with control arteries. In fact, media stress (wall tension divided by media thickness) was similar in breast cancer feed arteries and control arteries when maximally stimulated with endothelin-1 (300 nmol/L, Fig. 3a) or elevated [K^+^]_o_ (80 mmol/L, Fig. 3b). In contrast, the reduced media stress in breast cancer feed arteries compared to control arteries when exposed to norepinephrine (30 μmol/L, Fig. 3c) or U46619 (3 μmol/L, Fig. 3d) demonstrates that additional, structure-independent, functional differences contribute to the lower contractile responses in cancer arteries.

### VSMC Ca^2+^ responses and depolarizations are reduced during norepinephrine stimulation

We next investigated cellular signaling events linking receptor activation in VSMCs to vasocontraction in order to determine why media stress in breast cancer feed arteries is lower than in control arteries during norepinephrine-induced contractions (Fig. 3c) but not during stimulation with endothelin-1 (Fig. 3a).

**Fig. 4:**
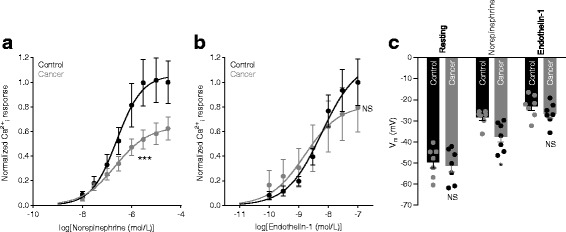
Norepinephrine-induced vascular smooth muscle cell (VSMC) intracellular Ca^2+^ responses and membrane depolarizations are reduced in breast cancer feed arteries compared to control arteries. **a, b** Normalized VSMC intracellular Ca^2+^ responses during stimulation with norepinephrine (**a**) (*n* = 7) or endothelin-1 (**b**) (*n* = 6). Curves are results of least-squares fits to sigmoidal functions and compared using extra-sum-of-squares *F* tests. **c** VSMC membrane potentials (n = 7) at rest and in the presence of 10 μmol/L norepinephrine or 100 nmol/L endothelin-1. Data were compared by repeated measures two-way analysis of variance followed by Sidak’s post-hoc test. *P < 0.05, ***P < 0.001, NS: not significantly different *vs.* control

We observed no difference in VSMC baseline intracellular Ca^2+^ before application of agonists: the Fura-2 fluorescence ratio in breast cancer feed arteries was 98.9 ± 6.3% of that in control arteries (*n* = 7, *P* = 0.60, paired two-tailed Student’s *t* test). Consistent with lower media stress in response to norepinephrine (Fig. 3c), the norepinephrine-induced increase in VSMC intracellular [Ca^2+^] was attenuated in breast cancer feed arteries compared to control arteries (Fig. 4a). The VSMC intracellular Ca^2+^ response to endothelin-1 did not similarly differ between breast cancer feed arteries and control arteries (Fig. 4b), which is in line with the equivalent levels of endothelin-1-induced media stress (Fig. 3a).

As shown in Fig. 4c, VSMC resting membrane potentials did not differ between cancer and control arteries whereas norepinephrine-induced depolarization was significantly smaller in VSMCs of breast cancer feed arteries (ΔV_m_ = 14.5 mV) than control arteries (ΔV_m_ = 25.2 mV). In contrast, depolarization in response to endothelin-1 was very similar in VSMCs of breast cancer feed arteries (ΔV_m_ = 24.0 mV) and control arteries (ΔV_m_ = 24.2 mV). The difference in agonist dependency between membrane potential responses of breast cancer feed arteries and control arteries was confirmed statistically by significant interaction (*P* < 0.05) in two-way ANOVA.

### Expression of α_1A_-adrenoceptors is reduced in cancer feed arteries

**Fig. 5:**
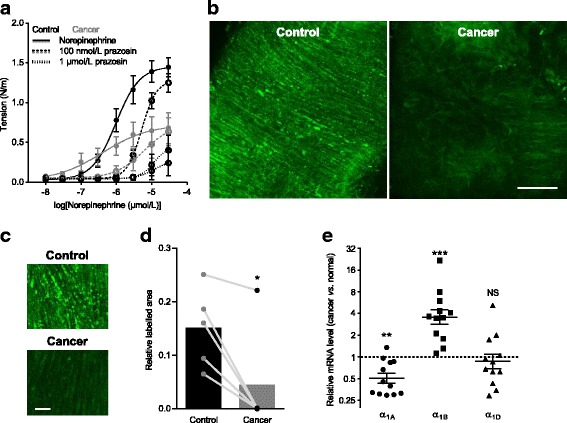
Expression of α_1A_-adrenoceptors is reduced in breast cancer feed arteries compared to matched control arteries. **a** Concentration-dependent inhibition of norepinephrine-induced vasocontraction by the α_1_-adrenoceptor antagonist prazosin (n = 6). The curves are the results of least-squares fits to sigmoidal functions. **b**, **c** Original images of labelling achieved with 1 μmol/L BODIPY™ FL prazosin in control arteries and breast cancer feed arteries. The second image within tunica media—identified relative to the autofluorescent internal elastic lamina—was extracted from Z-stack series (1-μm steps) and used for quantification. Low (**b**) (scale bar 50 μm) and high (**c**) (scale bar 10 μm) magnification images are shown. **c** The light intensity is equally enhanced for control and cancer feed arteries in order to make the autofluorescence of vascular smooth muscle cells (VSMCs) visible and confirm the stronger prazosin binding to VSMCs in control arteries compared to cancer feed arteries. **d** Relative fluorescence-labelled areas in the arterial media of breast cancer feed arteries and matched control arteries (*n* = 5). Data were compared by the paired two-tailed Student’s *t* test. **e** Expression of messenger RNA (mRNA) for α_1_-adrenoceptors in breast cancer feed arteries relative to matched control arteries (*n* = 12). The expression levels for the α_1_-adrenoceptors were normalized to the expression of the transferrin receptor. Distributions were compared to a hypothetical mean of 1 by the one-sample Student’s *t* test. *P < 0.05, **P < 0.01, ***P < 0.001, NS: not significantly different *vs.* control or as indicated

To further explore the molecular background for the attenuated norepinephrine-induced media stress, intracellular Ca^2+^ responses, and VSMC depolarization, we next investigated the involvement of α_1_-adrenoceptors. As expected, norepinephrine-induced vasocontraction was concentration-dependently abrogated by the α_1_-adrenoceptor antagonist prazosin (Fig. 5a). Because near-complete inhibition of norepinephrine-induced vasocontraction required 1 μmol/L prazosin (Fig. 5a), we next used this concentration of fluorescent BODIPY™-labelled prazosin to evaluate VSMC expression of α_1_-adrenoceptors (Fig. 5b): in support of lower α_1_-adrenoceptor expression, labelling with BODIPY™ FL prazosin was very strongly reduced in tunica media of breast cancer feed arteries compared to control arteries (Fig. 5b-d). The confocal planes of the quantified images were adjusted relative to the internal elastic lamina in order to make sure that they were at comparable levels in the tunica media.

We also investigated expression of α_1_-adrenoceptors at messenger RNA level and found a specific decrease in α_1A_-adrenoceptor expression in breast cancer feed arteries compared with control arteries (Fig. 5d). In contrast, messenger RNA levels for α_1D_-adrenoceptors were unchanged and expression of α_1B_-adrenoceptors was paradoxically increased in breast cancer feed arteries (Fig. 5d).

### Sympathetic vasocontraction is attenuated in cancer feed arteries

**Fig. 6:**
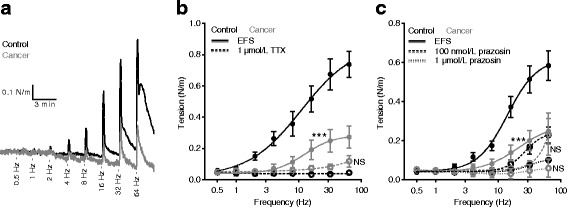
Arterial contractions in response to electrical field stimulation of perivascular sympathetic nerve endings are reduced in breast cancer feed arteries compared to matched control arteries. **a** Original traces showing contractions of breast cancer feed arteries and control arteries elicited by electrical field stimulation. **b**, **c** Concentration-response curves showing frequency-dependent contractions of breast cancer feed arteries and control arteries (n = 5–6) in response to electrical field stimulation (EFS) (**b**, **c**) and their inhibition by tetrodotoxin (TTX) (**b**) and prazosin (**c**). The curves are the results of least-squares fits to sigmoidal functions, and we compared them using the extra-sum-of-squares *F* test. ***P < 0.001. NS: not significantly different *vs.* control under similar conditions

Because norepinephrine is an important sympathetic neurotransmitter, we next explored whether contractile responses to electrical field stimulation of perivascular nerves were reduced in breast cancer feed arteries compared to control arteries (Fig. 6a-c). First, we established stimulation parameters that ensured complete tetrodotoxin (TTX) sensitivity of the vasocontractile response (Fig. 6b). Then, we showed that the TTX-sensitive responses were strongly attenuated in breast cancer feed arteries compared to control arteries (Fig. 6a-c), and finally we confirmed that the vasocontractile responses to perivascular nerve stimulation were completely prevented by 1 μmol/L prazosin (Fig. 6c). Prazosin inhibited vasocontraction in response to electrical field stimulation (Fig. 6c) at concentrations similar to those inhibiting contractions elicited by exogenously applied norepinephrine (Fig. 5a).

### Vasorelaxant function is preserved in norepinephrine-pre-contracted cancer feed arteries

**Fig. 7:**
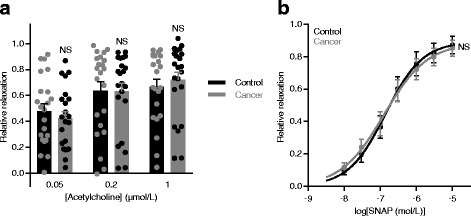
Endothelium-dependent and endothelium-independent relaxations are unaffected in norepinephrine-pre-contracted breast cancer feed arteries compared to matched control arteries. Relaxation of norepinephrine-pre-contracted arteries in response to acetylcholine (**a**) (*n* = 22) and nitric oxide (NO)-donor *S*-nitroso-*N*-acetyl-DL-penicillamine) (SNAP) (**b**) (n = 7). **a** Data were compared by repeated measures two-way analysis of variance followed by Bonferroni’s post-hoc test. **b** Curves are results of least-squares fits to sigmoidal functions and were compared using the extra-sum-of-squares *F* test. NS: not significantly different *vs.* control

Vasocontraction develops under the influence of endothelium-derived vasoactive substances. Therefore, we next studied endothelial function in order to evaluate if attenuated vasocontraction of cancer feed arteries is explained by changes in endothelium-dependent vasorelaxation. When arteries were pre-contracted with norepinephrine, no difference in the response to acetylcholine (Fig. 7a) or nitric oxide (NO)-donor *S*-nitroso-*N*-acetyl-DL-penicillamine (SNAP, Fig. 7b) was observed between breast cancer feed arteries and control arteries.

On this basis, we conclude that reduced vasocontractile responses of breast cancer feed arteries (Figs. 1 and 6, and Additional file 1: Figure S1) are explained by thinner wall structure (Fig. 2) and reduced VSMC receptor expression (Fig. 5) and not by differences in endothelial function (Fig. 7).

## Discussion

We show here that murine breast cancer feed arteries are functionally and structurally abnormal: compared to similarly-sized control arteries from equivalent anatomical locations along the mammary lines, vasocontractile responses of breast cancer feed arteries are reduced (Fig. 1 and Additional file 1: Figure S1) because of (a) structural thinning of tunica media due to fewer VSMC layers (Fig. 2) and (b) reduced activation during norepinephrine stimulation (Fig. 3) explained by diminished α_1A_-adrenoceptor expression (Fig. 5), leading to smaller VSMC depolarizations (Fig. 4c) and decreased VSMC intracellular Ca^2+^ responses (Fig. 4a). In particular, our data document striking attenuation of sympathetic vasocontraction in response to endogenous norepinephrine released from perivascular nerve endings (Fig. 6).

We observed overall reduction in α_1_-adrenoceptor expression in VSMCs of breast cancer feed arteries—evidenced by attenuated binding of the α_1_-adrenoceptor antagonist prazosin in the tunica media (Fig. 5b-d)—and confirmed lower levels of messenger RNA transcripts coding for α_1A_-adrenoceptors (Fig. 5e). Noteworthy, media stress in response to depolarization induced with elevated [K^+^]_o_ was unaffected in breast cancer feed arteries compared to control arteries (Fig. 3b). Endothelin-1-induced VSMC depolarization, VSMC intracellular Ca^2+^ dynamics, and media stress also did not differ between breast cancer feed arteries and control arteries (Fig. 3a and 4b, c). These findings support the concept that apart from differences in receptor expression, individual VSMCs of breast cancer feed arteries are functionally unaffected. Our structural evaluation of the arterial media assumes that it consists of uniformly sized VSMCs without infiltration from non-contractile—e.g., fibroblastic—cells, and this assumption is supported by the equivalent media stress in cancer feed arteries and control arteries depolarized with elevated extracellular [K^+^] (Fig. 3b).

Reduced norepinephrine-induced vasoconstriction of breast cancer feed arteries—which have similar relaxed diameters as control arteries—leads to lower vascular resistance and predictably increases perfusion of tumor tissue. Because of lower norepinephrine responses, we expect that tension development in breast cancer feed arteries is more disconnected from systemic cardiovascular regulation than in control arteries, and we predict a strong influence from local metabolic and paracrine factors. Indeed, our findings support that vasomotor activity in response to locally accumulated vasodilator metabolites (e.g., H^+^ (Additional file 1: Figure S1)) and paracrine signaling substances (e.g., endothelin-1 (Fig. 1c and Additional file 1: Figure S1B)) is preserved in breast cancer feed arteries. In accordance, studies show that endothelin-1 is important for myogenic tone development in tumor arterioles [28].

Thinner tunica media of breast cancer feed arteries compared to control arteries is explained by fewer VSMCs per unit artery length resulting in fewer VSMC layers (Fig. 2j, k). Sizes of individual VSMCs and the volume fraction of VSMCs in tunica media did not differ between breast cancer feed arteries and control arteries (Fig. 2f-i). Possible explanations for altered arterial structure and function include increased mechanical forces due to reduced downstream resistance. Disturbed flow patterns regulate arterial structure with high shear stress generally leading to outward remodeling and low shear stress causing inward remodeling [29, 30]. In particular, the blood flow patterns modulate vessel growth during vascular endothelial growth factor-induced angiogenesis [31]. Altered biochemical composition of the extracellular tumor microenvironment may also influence the structure and function of breast cancer feed arteries. Disturbed local acid-base conditions of cancer tissue have potential to modify arterial remodeling [32]: recent evidence supports that intracellular pH influences VSMC proliferation whereas local pH gradients along VSMC protrusions modify VSMC migration [29]. Altered pH_o_ conditions have also been shown to regulate, for instance, cell-cell and cell-matrix adhesion [33, 34]. Therefore, acid-base disturbances are powerful signals predicted to impact arterial structure development. The altered wall structure was present even in arteries from the smallest investigated tumors (Fig. 2d), suggesting that the change in arterial development or adaptation of arterial structure occurs early in cancer development. The specialization of the cancer feed arteries towards reduced resistance likely facilitates oxygen and substrate delivery during carcinogenesis and early tumor expansion, which emphasizes its potential as a therapeutic target.

Systemic application of vasoactive substances leads to heterogeneous changes in vascular resistance between different organ systems and often influences perfusion pressure. In addition to the effects of cardiac function, perfusion pressure is a function of total peripheral resistance. As a consequence, tumor perfusion depends on changes in the resistance of the tumor vasculature relative to other vascular beds. Considering the diminished vasocontractile response of breast cancer feed arteries, increased sympathetic tone is predicted to increase perfusion pressure, have marginal effects on tumor vascular resistance, and ultimately increase tumor perfusion. Reduced precapillary resistance due to attenuated sympathicus-mediated vasoconstriction will also increase capillary pressure and fluid filtration and likely contribute to edema formation in tumor tissue. Thus, our results indicate that variation in surface receptor expression between cancer and normal arteries can be utilized to change resistance of the tumor vasculature relative to the peripheral vasculature as a whole and thereby modify oxygen and nutrient delivery to cancer tissue. As suggested for bradykinin signaling in tumor arteries, a relative decrease in tumor vascular resistance can facilitate chemotherapy delivery [35, 36].

## Conclusions

We showed that breast cancer feed arteries structurally and functionally differ from normal blood vessels. Structural thinning and lower α_1A_-adrenoceptor expression in breast cancer feed arteries give rise to attenuated vasocontraction particularly in response to norepinephrine released from perivascular sympathetic nerves. We propose that functional specialization of breast cancer arteries can be exploited to modify tumor perfusion and thereby local delivery of drugs, nutrients, and oxygen or for boosting treatment responses to chemotherapy and radiotherapy.

## Additional file

Additional file 1: **Figure S1.** Vasocontraction is attenuated in breast cancer feed arteries compared to matched control arteries under acidic conditions. Concentration-dependent contractions in response to norepinephrine (A), endothelin-1 (B), and elevated [K^+^]_o_ (C) at pH_o_ 6.8 (*n* = 8). For norepinephrine and K^+^, contractions elicited in the same arteries at pH_o_ 7.4 are shown for comparison. Because of slow relaxation of arteries after washout of endothelin-1, vasocontraction to endothelin-1 was only tested at one level of pH_o_ for each artery. Experiments with elevated [K^+^]_o_ were performed in presence of 1 μmol/L phentolamine in order to avoid effects of norepinephrine released from sympathetic nerve endings in response to depolarization. Curves are results of least-squares fits to sigmoidal functions and compared using extra-sum-of-squares *F* tests. **P < 0.01, ***P < 0.001 *vs.* control, both at pH_o_ 6.8. (PDF 244 kb)

## Abbreviations

ANOVA: Analysis of variance
EDTA: Ethylenediaminetetraacetic acid
o: Extracellular
PSS: Physiological saline solution
SNAP: *S*-nitroso-*N*-acetyl-DL-penicillamine
TTX: Tetrodotoxin
V_m_: Membrane potential
VSMC: Vascular smooth muscle cell
