# Supplementary material for: Murine breast cancer feed arteries are thin-walled with reduced α1A-adrenoceptor expression and attenuated sympathetic vasocontraction
Source: Breast Cancer Res. 2018 Mar 22;20:20. doi: 10.1186/s13058-018-0952-8 (PMC5863844; doi:10.1186/s13058-018-0952-8)
Supplement: Supplementary file 1 — Figure S1. Vasocontraction is attenuated in breast cancer feed arteries compared to matched control arteries under acidic conditions. Concentration-dependent contractions in response to norepinephrine (A), endothelin-1 (B), and elevated [K+]o (C) at pHo 6.8 (n = 8). For norepinephrine and K+, contractions elicited in the same arteries at pHo 7.4 are shown for comparison. Because of slow relaxation of arteries after washout of endothelin-1, vasocontraction to endothelin-1 was only tested at one level of pHo for each artery. Experiments with elevated [K+]o were performed in presence of 1 μmol/L phentolamine in order to avoid effects of norepinephrine released from sympathetic nerve endings in response to depolarization. Curves are results of least-squares fits to sigmoidal functions and compared using extra-sum-of-squares F tests. **P < 0.01, ***P < 0.001 vs. control, both at pHo 6.8. (PDF 244 kb) [file 13058_2018_952_MOESM1_ESM.pdf]

Additional data for:

Murine breast cancer feed arteries are thin-walled with reduced α_{1A}-adrenoceptor expression and attenuated sympathetic vasocontraction

Anne Sofie Froelunde, Marit Ohlenbusch, Kristoffer B. Hansen, Nicolai Jessen, Sukhan Kim, Ebbe Boedtkjer

Department of Biomedicine, Aarhus University, Denmark

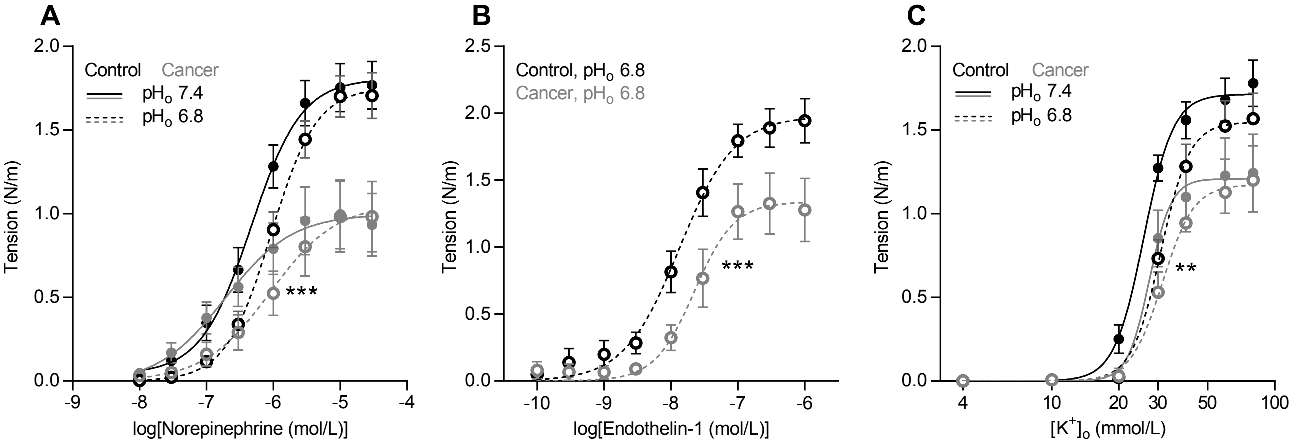

Additional Figure S1. Vasocontraction is attenuated in breast cancer feed arteries compared to matched control arteries under acidic conditions. Concentration-dependent contractions in response to norepinephrine (**A**), endothelin-1 (**B**), and elevated $[K^+]_o$ (**C**) at pH_o 6.8 (n=8). For norepinephrine and K^+, contractions elicited in the same arteries at pH_o 7.4 are shown for comparison. Because of slow relaxation of arteries after washout of endothelin-1, vasocontraction to endothelin-1 was only tested at one level of pH_o for each artery. Experiments with elevated $[K^+]_o$ were performed in presence of 1 μmol/L phentolamine. Curves are results of least-squares fits to sigmoidal functions and compared using extra sum-of-squares *F*-tests. $^{**}P<0.01$, $^{***}P<0.001$ *vs.* Control, both at pH_o 6.8.
